# Ventricular fibrillation caused by massive right coronary air embolism: a case report

**DOI:** 10.1186/s43044-024-00592-1

**Published:** 2025-01-06

**Authors:** Hongcai Zhang, Ai-ling Huang, Qian Nie, Haseeb Sattar, Xie Wen

**Affiliations:** 1 Department of Cardiology, Hospital of Chengdu University of Traditional Chinese Medicine, Chengdu, Sichuan Province People’s Republic of China; 2 Department of Cardiology, Hospital of Pidu District of Traditional Chinese Medicine, Chengdu, Sichuan Province People’s Republic of China; 3 Department of Medicine, Urban Vocational College of Sichuan, Chengdu, China

**Keywords:** Right coronary artery, Percutaneous coronary intervention, Fractional flow reserve, Coronary artery air embolism, Coronary slow flow

## Abstract

**Background:**

Coronary air embolism is a rare but severe complication of coronary interventions.

**Case presentation:**

We present a case of a massive air embolism in the right coronary artery during percutaneous coronary intervention, resulting in ventricular fibrillation. The patient was successfully resuscitated with electric defibrillation, leading to full recovery and TIMI 3 coronary flow. The final fractional flow reserve showed no residual coronary microvascular dysfunction.

**Conclusion:**

Our finding suggests that inducing strong myocardial contractions with a cardioverter defibrillator may effectively disperse large air emboli and restore coronary circulation.

## Background

During PCI, coronary artery air embolism (CAE) may arise as a rare form of life-threatening complication which is mostly iatrogenic. In the literature, few cases of isolated CAE have been reported without proper clinical management guidelines, especially during the PCI [1]. This study aims to explain the complications that can occur during the PCI and their prompt management, especially an air embolism.

## Case presentation

A 65-year-old male with refractory stable angina pectoris and a 40-year history of smoking and hypertension were admitted for coronary angiography (CAG). A critical proximal lesion in the RCA with an FFR of 0.65 was identified. During PCI, accidental air injection resulted in two large air emboli in the distal and proximal RCA, causing coronary slow flow (CSF) and ST-segment elevation in the inferior leads, complete atrioventricular block, and hypotension. The estimated volume of air inadvertently injected was approximately 2–3 mL. Despite administration of dopamine and 100% oxygen, the patient suffered VF and cardiac arrest. Emergency defibrillation successfully resuscitated the patient, restoring TIMI 3 flow in the RCA without vascular complications. A drug-eluting stent was then implanted, and the final FFR was 0.93. The patient was reevaluated postoperatively. Transthoracic echocardiography (TTE) revealed 55% of left ventricular ejection fraction (LVEF) without any evidence of thrombi and regional wall abnormalities. During hospitalization, the patient remained hemodynamically stable and was later discharged after two days.

## Discussion

CAE, although rare, is a recognized complication of PCI, often due to operator error. Air emboli typically appear as “mobile spherical” or “bead-like” structures on angiography, often accompanied by CSF [2]. Small air amounts are metabolized naturally, but larger emboli can obstruct coronary circulation and cause significant clinical complications, including death. Treatments for CAE include oxygen therapy to increase oxygen saturation, which helps dissolve nitrogen in the embolus, mechanical perfusion to aspirate or displace the air, and pharmacologic interventions like vasopressors or atropine [3, 4]. In this case, VF likely resulted from the combined effects of dopamine-induced vasoconstriction and air embolism exacerbating ischemia. The use of electric defibrillation not only restored cardiac rhythm but may have also mechanically dispersed the embolus, facilitating rapid resolution and preventing further microvascular damage, and the patient did not experience any further arrhythmia during hospitalization.

## Conclusion

**Fig. 1:**
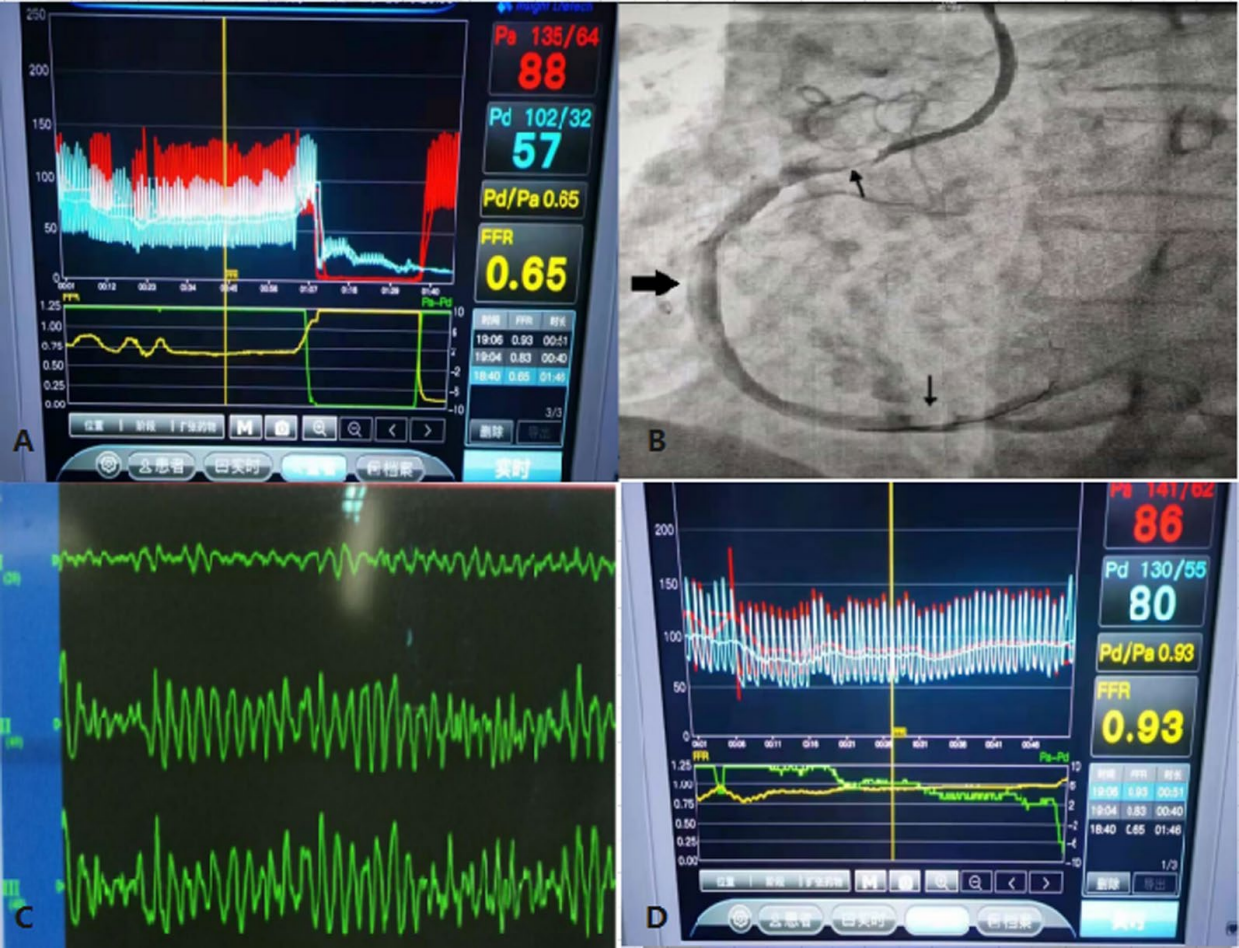
**A** FFR before PCI.** B** During angiography, accidental injection caused two air bubbles (black arrows) located in distal and proximal segments which led to occlusion of the right coronary.** C** Monitor recorded ventricular fibrillation.** D** FFR post-stent implanted.

Unintended air embolism during coronary procedures can lead to severe outcomes. Defibrillation-induced myocardial contractions might be an effective emergency intervention for large CAE, warranting further investigation (Fig. 1).

## Data Availability

The related data and material will be provided on request.

## Abbreviations

RCA: Right coronary artery
PCI: Percutaneous coronary intervention
FFR: Fractional flow reserve
CAE: Coronary artery air embolism
CSF: Coronary slow flow
CAG: Coronary angiography

## References

[1] Kariyanna PT et al. (2018) Coronary air embolism during coronary angiography: a systematic review. SciFed J Cardiol 2

[2] Khan M (1995) Coronary air embolism: incidence, severity, and suggested approaches to treatment. Cathet Cardiovasc Diagn 36(4):313–318 8719380 10.1002/ccd.1810360406

[3] Malik N et al (2017) Air embolism: diagnosis and management. Future Cardiol 13(4):365–378 28644058 10.2217/fca-2017-0015

[4] Suastika LOS, Oktaviono YH (2016) Multiple air embolism during coronary angiography: How do we deal with it? Clin Med Insights: Cardiol 10:CMC.S38040 10.4137/CMC.S38040 PMC4874743 27226738

